# Dopamine, Alpha-Synuclein, and Mitochondrial Dysfunctions in Parkinsonian Eyes

**DOI:** 10.3389/fnins.2020.567129

**Published:** 2020-10-19

**Authors:** Alessia Indrieri, Rocco Pizzarelli, Brunella Franco, Elvira De Leonibus

**Affiliations:** ^1^Telethon Institute of Genetics and Medicine, Pozzuoli, Italy; ^2^Institute for Genetic and Biomedical Research, National Research Council, Milan, Italy; ^3^Center for Life Nanoscience, Istituto Italiano di Tecnologia, Rome, Italy; ^4^Medical Genetics, Department of Translational Medical Science, University of Naples “Federico II”, Naples, Italy; ^5^Institute of Biochemistry and Cellular Biology, National Research Council, Rome, Italy

**Keywords:** Parkinson’ disease, retina, mitochondria, visual dysfunctions, alpha-synuclein, dopamine, parkinsonism, optic neuropathies

## Abstract

Parkinson’s disease (PD) is characterized by motor dysfunctions including bradykinesia, tremor at rest and motor instability. These symptoms are associated with the progressive degeneration of dopaminergic neurons originating in the *substantia nigra pars compacta* and projecting to the corpus striatum, and by accumulation of cytoplasmic inclusions mainly consisting of aggregated alpha-synuclein, called Lewy bodies. PD is a complex, multifactorial disorder and its pathogenesis involves multiple pathways and mechanisms such as α-synuclein proteostasis, mitochondrial function, oxidative stress, calcium homeostasis, axonal transport, and neuroinflammation. Motor symptoms manifest when there is already an extensive dopamine denervation. There is therefore an urgent need for early biomarkers to apply disease-modifying therapeutic strategies. Visual defects and retinal abnormalities, including decreased visual acuity, abnormal spatial contrast sensitivity, color vision defects, or deficits in more complex visual tasks are present in the majority of PD patients. They are being considered for early diagnosis together with retinal imaging techniques are being considered as non-invasive biomarkers for PD. Dopaminergic cells can be found in the retina in a subpopulation of amacrine cells; however, the molecular mechanisms leading to visual deficits observed in PD patients are still largely unknown. This review provides a comprehensive analysis of the retinal abnormalities observed in PD patients and animal models and of the molecular mechanisms underlying neurodegeneration in parkinsonian eyes. We will review the role of α-synuclein aggregates in the retina pathology and/or in the onset of visual symptoms in PD suggesting that α-synuclein aggregates are harmful for the retina as well as for the brain. Moreover, we will summarize experimental evidence suggesting that the optic nerve pathology observed in PD resembles that seen in mitochondrial optic neuropathies highlighting the possible involvement of mitochondrial abnormalities in the development of PD visual defects. We finally propose that the eye may be considered as a complementary experimental model to identify possible novel disease’ pathways or to test novel therapeutic approaches for PD.

## Introduction

Parkinson’s disease (PD) represents the second most common neurodegenerative disorder after Alzheimer’s disease. The prevalence of this condition in industrialized countries is generally estimated at 1% in people over 60 years of age representing an important burden for health systems considering that to date treatment options are mostly symptomatic (de Lau and Breteler, 2006; Kalia and Lang, 2015; Rodriguez-Blazquez et al., 2015). PD is a complex neurodegenerative disorder and the etiology of the disease is unknown in most patients. Risk factors include age, male gender and some environmental factors. Moreover, different genetic causes have been identified and both rare and common genetic variants contribute to disease risk, onset, and progression. To date, mutations in more than 20 genes, most of which are highly penetrant and often cause early onset or atypical symptoms, have been associated with the disease (reviewed in Puschmann, 2017; Deng et al., 2018; Blauwendraat et al., 2019) (Table 1).

**TABLE 1:** Monogenic causes of Parkinson’s disease.

**Gene**	**Protein**	**Mutation**	**Inheritance**	**Function**	**Proposed disease mechanism**
*SNCA*	α-Synuclein	Missense or multiplication	Dominant	Presynaptic signaling and membrane trafficking.	Gain of function or overexpression
*LRRK2*	Leucine-rich repeat serine/threonine-protein kinase 2	Missense	Dominant	Neuronal plasticity, autophagy, and vesicle trafficking. 10% located in the outer mitochondrial membrane	Gain of function
*PRKN*	Parkin	Missense, exon deletion or duplication	Recessive	Proteasomal degradation, mitophagy, cell death, oxidative stress	Loss of function
*PINK1*	PTEN induced kinase 1	Missense, deletion	Recessive	Protection against mitochondrial dysfunction, mitophagy	Loss of function
*POLG*	DNA polymerase subunit gamma-1	Missense	Dominant/recessive	Replication of mitochondrial DNA	Loss of function
*PARK7*	DJ-1	Missense	Recessive	Cell death, oxidative stress	Loss of function
*ATP13A2*	Cation-transporting ATPase 13A2	Missense	Recessive	Lysosome and mitochondrial maintenance	Loss of function
*GBA*	Lysosomal acid glucosylceramidase	Missense	Dominant (incomplete penetrance)	Ceramide formation, glycolipid metabolism, turnover of cellular membranes	Likely loss of function
*FBX07*	F-box protein 7	Missense	Recessive	Proteasomal degradation, mitophagy, cell death, oxidative stress	Loss of function
*PLA2G6*	Phospholipase A2 group VI	Missense	Recessive	Phospholipid remodeling, arachidonic acid release, leukotriene and prostaglandin synthesis, cell death	Loss of function
*VPS35*	Vacuolar protein sorting-associated protein 35	Missense	Dominant	Transport of proteins from endosomes to the *trans-*Golgi network	Loss of function
*VPS13C*	Vacuolar protein sorting-associated protein 13C	Missense, deletion	Recessive	Mitochondrial function, maintenance of mitochondrial transmembrane potential, mitophagy, Golgi to endosome transport	Loss of function
*SYNJ1*	Synaptojanin 1	Missense	Recessive	Polyphosphoinositide phosphatase involved in clathrin-coated pit and synaptic vesicle dynamics	Loss of function
*DNAJC6*	Auxilin	Missense	Recessive	Clathrin-mediated endocytosis	Loss of function
*DNAJC13*	DnaJ heat shock protein family member C13	Missense	Dominant	Clathrin-mediated endocytosis, post-endocytic transport	Unclear
*TMEM230*	Transmembrane protein 230	Missense, deletion	Dominant	Trafficking and recycling of synaptic vesicles	Likely loss of function
*TWNK*	Twinkle mtDNA helicase	Missense	Dominant	mtDNA replication	Loss of function
*UCHL1*	Ubiquitin carboxyl-terminal hydrolase isozyme L1	Missense	Dominant	Processing of ubiquitin precursors and of ubiquitinated proteins	Likely loss of function
*HTRA2*	Serine protease HTRA2, mitochondrial	Missense	Recessive	Mitochondrial-dependent cell death	Unclear
*EIF4G1*	Eukaryotic translation initiation factor 4 gamma 1	Missense	Dominant	Component of the eIF4F complex, translation initiation	Unclear
*GIGYF2*	GRB10-interacting GYF protein 2	Missense	Dominant	Component of the 4EHP-GYF2 complex, repressor of translation initiation	Unclear
*CHCHD2*	Coiled-coil-helix-coiled-coil-helix domain containing 2, mitochondrial	Missense	Dominant	OHPHOS, mitochondrial-dependent cell death	Likely loss of function
					

Although genetic forms of PD represent a small fraction of all cases, they have provided important clues to the neuropathology of PD defining crucial underlining pathways such as α-synuclein (α-Syn) proteostasis, mitochondrial function, oxidative stress, calcium homeostasis, axonal transport, and neuroinflammation. Of note, many PD mutations affect the *SNCA* gene that encodes α-Syn and genes associated with mitochondrial function (Table 1).

PD is characterized by the death of dopamine (DA) neurons localized in the *substantia nigra pars compacta* (SNpc), from which neurons project to the corpus striatum and to other brain regions of the mesocorticolimbic system, and by the accumulation of cytoplasmic inclusions, called Lewy bodies, mainly consisting of aggregated α-Syn and ubiquitin (Spillantini et al., 1997).

The loss of nigro-striatal DA function leads to the classical parkinsonian motor symptoms including bradykinesia, tremor at rest, and postural and gait instability (Kalia and Lang, 2015; Postuma et al., 2015). The relationship between α-Syn aggregates and DA neuronal loss is the focus of several experimental studies; most of them convergently show that overexpression or mutation of the *SNCA* gene leads to the formation of α-Syn aggregates (oligomers and fibrils), which induce a time-dependent loss of DA neurons (Giordano et al., 2018). Lewy bodies are found not only in DA neurons, but are also present in different central nervous system (CNS) regions, and they are thought to contribute to other PD symptoms such as cognitive deficits, depression, sleep disorders, constipation, olfactory dysfunction, and visual defects (Braak et al., 2004; Mahlknecht et al., 2015), generally referred to as non-motor symptoms. Non-motor features are frequently observed in PD before the onset of the classical motor symptoms (Postuma et al., 2012). Recently, much attention has been given to these early non-motor symptoms as they represent a potential temporal window during which disease-modifying therapies acting to prevent or delay neurodegeneration could be administered (Siderowf and Lang, 2012; Kalia and Lang, 2015). The molecular mechanisms underlying non-motor symptoms in PD are being investigated in experimental and clinical studies, and one of the most dominant views is that proposed by Braak (Guo et al., 2018). According to Braak’s staging theory, this premotor or prodromal phase can precede the onset of classical parkinsonian motor symptoms by decades and it is due to the spreading of α-Syn pathology from ventral to dorsal brain regions (Braak et al., 2003, 2004). This hypothesis is supported by experimental evidence showing that α-Syn fibrils can spread across brain regions and more recent findings show that they can move from the gut to the brain (Challis et al., 2020). According to this hypothesis the eyes should be one of the first organs affected if fibrils spread from the nose, as previously suggested (Choudhry and Perlmuter, 2017), which would be in line with clinical and pre-clinical findings reporting specific visual symptoms in early stages of PD or dementia with Lewy bodies (DLB) (Armstrong, 2015; Himmelberg et al., 2018; Chung et al., 2019).

Among non-motor symptoms, one of the clinical manifestations present in the majority of PD patients is the presence of vision impairment and retinal abnormalities, including decreased visual acuity, abnormal spatial contrast sensitivity, color vision defects, or deficits in more complex visual tasks (Postuma et al., 2012; Guo et al., 2018). Moreover, accumulating experimental evidence suggests that pathological α-Syn aggregates are present in the retina and/or visual system of PD patients and of PD animal models. In addition, phosphorylated α-Syn accumulates in the retina as well as in the brain also at early stages, preceding the appearance of clinical signs of parkinsonism or dementia (Guo et al., 2018; Ortuno-Lizaran et al., 2018; Veys et al., 2019). Visual symptoms are probably the least invalidating symptoms in PD and DLB but they are being seriously considered as significant disease biomarkers. Given that the retina is an easy-access window to the pathological processes that are ongoing in the brain, visual tests and/or retinal imaging techniques are being developed to consider α-Syn aggregates in the eye as non-invasive biomarkers for PD (Weil et al., 2016; Guo et al., 2018; Ortuno-Lizaran et al., 2018).

The data reporting visual and retinal abnormalities in PD patients have been extensively reviewed recently (Weil et al., 2016; Guo et al., 2018). In this review we will integrate the clinical evidence with those reported in animal models highlighting the molecular mechanisms underlying neurodegeneration in parkinsonian eyes. Moreover, we will provide evidence about molecular disease pathways that are common to PD and mitochondrial diseases. We propose the retina not only as a site for early PD biomarkers identification through non-invasive approaches, but also as a powerful model containing most of the cellular subtypes involved in PD pathology, which can be used as a complementary tool to study disease’ pathways or to test novel therapeutic approaches for PD.

## Neuronal Substrates of Visual Symptoms in PD Patients

Ocular defects have been reported in about 80% of PD patients (Guo et al., 2018). Oculo-visual abnormalities include defects in primary vision such as visual acuity (Jones et al., 1992; Matsui et al., 2006; Archibald et al., 2011b), spatial contrast sensitivity (Bodis-Wollner et al., 1987; Bodis-Wollner, 2013), color vision (Price et al., 1992; Haug et al., 1994; Silva et al., 2005; Sartucci and Porciatti, 2006), eye movement (Winograd-Gurvich et al., 2006), or deficits in more complex visual tasks such as the perception of the spatial relationships between objects and visual hallucinations (Holroyd et al., 2001; Possin, 2010; Armstrong, 2011, 2015) (Table 2).

**TABLE 2:** Visual abnormalities in PD patients.

**Visual defect**	**Frequency in PD patients**	**Onset**	**References**
Visual acuity	30%	N/A	Jones et al., 1992; Holroyd et al., 2001; Matsui et al., 2006; Archibald et al., 2011a; Lin et al., 2015; Weil et al., 2016
Contrast sensitivity	N/A	Prodromal stage	Kupersmith et al., 1982; Bodis-Wollner et al., 1987; Bulens et al., 1987; Bodis-Wollner, 1988, 2013; Postuma et al., 2011; Stenc Bradvica et al., 2015; Weil et al., 2016
Color recognition	30–50%	Prodromal stage, controversial	Price et al., 1992; Buttner et al., 1994, 1995; Haug et al., 1994, 1995; Pieri et al., 2000; Muller et al., 2002; Silva et al., 2005; Sartucci and Porciatti, 2006; Cardoso et al., 2010; Postuma et al., 2011; Bertrand et al., 2012; Piro et al., 2014; Lin et al., 2015
Visual processing difficulties	10–30%	Prodromal stage	Postuma et al., 2011; Lin et al., 2015; Arrigo et al., 2017; Ekker et al., 2017
Object perception and recognition	70%	After motor symptoms	Montse et al., 2001; Muller et al., 2002; Uc et al., 2005; Gullett et al., 2013; Edelstyn et al., 2014; Weil et al., 2016
Visual hallucinations	20–40%	After motor symptoms	Fenelon et al., 2000; Diederich et al., 2005; Armstrong, 2015

**N/A, not available.**

Visual defects in PD may arise also as a consequence of cortical visual area defects. Thus, they can be attributed to changes at any level of the visual pathway as well as other sensory systems and motor function (Uc et al., 2005; Castelo-Branco et al., 2009). Histopathological and electrophysiological studies in humans and in experimental models suggest, however, that there are retina-specific visual defects in PD occurring in early stages of the pathology.

### Dopamine and α-Synuclein in the Context of Retina Cell Subtypes

**FIGURE 1:**
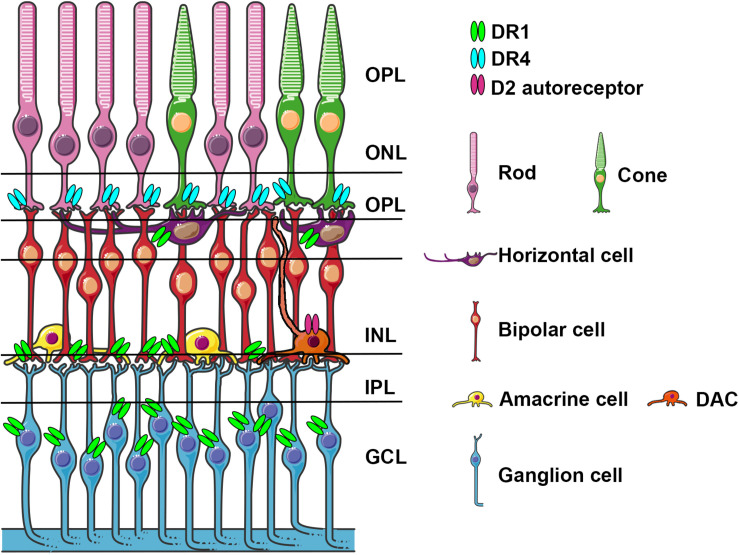
Schematic representation of the retina with cell types expressing specific dopamine receptors. Dopamine receptors D1R, D4R, and D2 autoreceptors localized on various cell types, are indicated light blue, light green and fuchsia, respectively. DACs stratify in the IPL and send axons like dendritic projections to OPL and to IPL.ONL, outer nuclear layer; OPL, outer plexiform layer; INL, inner nuclear layer; IPL, inner plexiform layer; GCL, canglion cell layer; DAC, dopaminergic amacrine cell.

There are six types of neurons and one type of glial cells (Müller glial cells) that constitute three cellular layers in the vertebrate retina: rod and cone photoreceptors in the outer nuclear layer (ONL), horizontal, bipolar, and amacrine interneurons and Müller glial cells in the inner nuclear layer (INL), and ganglion and displaced amacrine cells in the ganglion cell layer (GCL) (Figure 1). α-Syn aggregates have been identified in the retina of PD patients (Beach et al., 2014; Ortuno-Lizaran et al., 2018) and in particular in the GCL, INL and in the inner plexiform layer (IPL) that contains the synaptic contacts among and between bipolar, amacrine, and ganglion cells. Retinal photoreceptor cells capture light information and transmit it to RGC via bipolar and amacrine cells. Light information then arrives at the brain through the optic nerve formed by RGC axons.

Retinal cell types are connected and located according to a well-defined cytoarchitecture reflecting their functions in the transduction of visual *stimuli*: their heterogeneous nature makes this process rather complex from a physiological perspective (see Demb and Singer, 2015 for details). Rod photoreceptors are responsible for vision during low light conditions (scotopic vision). They contain rhodopsin, a G-Protein coupled receptor (GPCRs), as visual pigment and are particularly sensitive to light with a peak wavelength of ∼500 nanometers (nm). Cone photoreceptors are responsible for the vision during daylight (photopic vision) and are endowed with opsin as pigment. Contrary to rods, cones are involved in color perception and can be divided in three types according to the sensitivity exhibited for blue, red, and green light.

Once photoreceptors detect photons, a change in their membrane potential causes the neurotransmitter to be released onto bipolar cells (the only neuronal type spanning both the outer and inner retina) and horizontal cells. The latter are a class of inhibitory neurons fulfilling feed-back and feed-forward inhibition on both photoreceptors and bipolar cells. Downstream of bipolar cells, the electric signal is sent to RGCs whose axons form the optic nerve responsible for transmitting information to the brain. Intermingled among bipolar and RGC, amacrine cells can provide feed-forward and feed-back inhibition to both cell types by releasing γ-aminobutyric acid (GABA) and glycine. Muller cells, seeded with their bodies in the INL, are the main glial cells of the retina and support neuronal functions.

As in the brain, dopamine plays a key role also in the retina and is involved in a variety of processes such as modulation of light adaptation, color vision, retinal development, synaptic formation and transmission (Witkovsky, 2004; McMahon et al., 2014).

DA in the retina is synthetized in a subpopulation of amacrine cells (A18) localized in the IPL. Amacrine cells represent ∼35% of all cell types and they are classified based on the synthetized neurotransmitters, which mainly include glycine and GABA. Most DA amacrine cells (DACs) also synthetize GABA. DACs constitute only a small part of amacrine cells and they often co-express GABA and glycine (May et al., 2008; Roy and Field, 2019). DACs have long axon-like processes reaching the IPL, the GCL and sometimes the OPL and processes that overlap and branch forming a densely packed network of dendrites (Figure 1). Similar to what happens in the mesencephalon, DA is released in a tonic and phasic fashion and its action potential is influenced by AMPA glutamate receptor activation, GABA and glycine (Gustincich et al., 1997; Feigenspan et al., 1998). DA exerts its action through direct synaptic contacts and through volumetric diffusion. Given its long-distance and dense network it has the potential to influence the activity at different levels and on different cell subtypes in the retina (Witkovsky, 2004; Roy and Field, 2019). DA acts on five G-protein-coupled receptor subtypes grouped in D-like receptors (D1 and D5) and D2-like (D2, D3, D4) linked to the activation (D1-like) and inhibition (D2-like) of cyclic-AMP (Seeman and Van Tol, 1994; Witkovsky, 2004). In the retina, similar to what has been described in the mesencephalon, DACs contain D2 auto-receptors whose activation negatively influences DA release. In Figure 1 we have schematized the position of DA receptors in the different retina subtypes, which shows a cell type specific distribution, suggesting a functional dissociation between them. DA receptor subtypes differ in their sensitivity to DA (D5 > D2 > D1) allowing a differential recruitment during the day/light cycle characterized by high/low DA synthesis and release, respectively. This observation suggested that DA levels in the eye contribute to circadian rhythms and to a shift from rod-mediated to cone-mediated vision (Witkovsky, 2004). DA exerts its action in both outer and inner retinal cells; a detailed description of the underlying mechanisms goes beyond the scope of this review and have been extensively and elegantly addressed previously by Witkovsky (2004). Through pharmacological experiments, including studies using neurotoxins directly injected into the eye specifically acting on DA cells, it has been shown that DA improves spatial contrast detection and amplifies the cone pathway producing a shift from rod-dominant to cone-dominant vision during daylight (Dowling and Ehinger, 1975; Savy et al., 1995; Gustincich et al., 1999). Light stimuli activate DA cells, triggering DA release (Puopolo et al., 2001; Witkovsky, 2004); DA through the activation of D4R stimulation reduces the rod-cone communication, and through the activation of D1 receptors uncouples H1-type horizontal cells; both mechanisms increasing the direct response of cones in photopic conditions. By contrast, the absence of DA favors the rod-cone conductance and shunting of the cone electrical signal (Veruki and Wassle, 1996; Puopolo et al., 2001; Witkovsky, 2004; Roy and Field, 2019). Interestingly, a mouse model where the synthesis of DA was specifically prevented in the retina was generated by conditional inactivation of the tyrosine hydroxylase (TH) gene, which encodes the DA synthesis rate limiting enzyme. These mice showed deficits in light-adapted electroretinogram (ERG) responses, contrast sensitivity, acuity, and retinal circadian rhythms. These specific deficits could be mimicked in either DA, D1R, and D4R knock-out (KO) mice and rescued by D1R or D4R agonists (Jackson et al., 2012). In the retina of healthy individuals, the presence of endogenous α-Syn has been described in the GCL, IPL and INL, and also in photoreceptor outer segments and their terminals in the outer plexiform layer (OPL) (Martinez-Navarrete et al., 2007; Leger et al., 2011). These data are consistent with the presence of the α-Syn monomeric form in the cytoplasm and presynaptic nerve terminals and with its involvement in physiological functions such as synaptic plasticity, vesicle trafficking, and neurotransmission (Bendor et al., 2013).

Based on this set of experimental evidence highly specific DA-dependent vision deficits might be expected in PD patients. However, the visual impairment can be worsened by deficits related to α-Syn pathology and/or mitochondrial related dysfunction that, although might preferentially impair DA neurons function in the initial stage of the pathology, are also harmful for other cell types leading to widespread neurodegeneration and impaired visual function.

**FIGURE 2:**
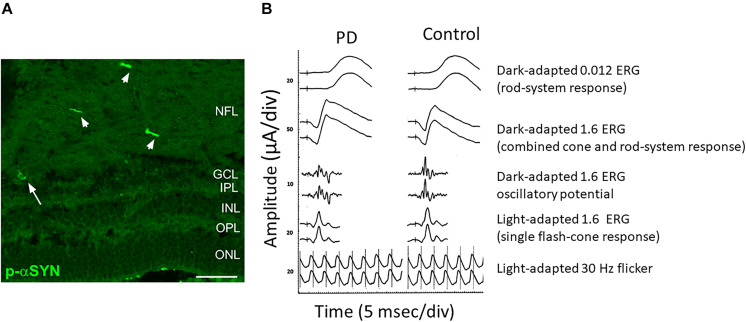
Anatomical and functional alterations in the retina of a PD patient. **(A)** Retinal section from a PD patient stained for Phospho-α-Synuclein shows the presence of aggregates both in neuronal axons (arrowheads) and soma (arrow). **(B)** Comparison of the scotopic a-wave, photopic b-wave and the Oscillatory Potential recorded from a control and a PD patient. Note the reduction in amplitude. Modified from Veys et al. (2019) **(A)** and Nowacka et al. (2015) **(B)** under terms of the Creative Commons Attribution 4.0 International License (http://creativecommons.org/licenses/by/4.0/).

As mentioned before, immunohistological studies on PD patients’ postmortem retinas revealed the presence of α-Syn aggregates and Lewy bodies in dispersed amacrine cells at the border of the INL and in the RGC layer, and diffuse α-Syn depositions and Lewy neurites in the IPL (Beach et al., 2014; Bodis-Wollner et al., 2014a). Different studies reported α-Syn aggregates/inclusions and phosphorylated α-Syn at serine 129 (pSer129-α-Syn) in the RGC, IPL and ONL but never in the outer retina in PD patients (Figure 2A). These α-Syn formations were not present in age-matched controls where the unphosphorylated α-Syn is seen in retinal cell layers (Beach et al., 2014; Bodis-Wollner et al., 2014a; Ho et al., 2014; Ortuno-Lizaran et al., 2018). As already shown in the brain, abnormal pSer129-α-Syn may play a key role in the control of α-Syn functions, aggregation, Lewy body formation, and neurotoxicity (Oueslati, 2016).

### Visual Deficits in PD Patients

Among the visual disfunctions described in PD patients, visual acuity, contrast sensitivity, and color vision impairment are more directly linked to the retinal alteration observed in PD patients showing α-Syn aggregation, retinal neuron degeneration and reduced retinal DA levels.

Visual acuity refers to the ability to discern the shapes and details. Defects in this task were reported in about 30% of PD patients and this effect is not corrected by DA (Jones et al., 1992; Matsui et al., 2006; Archibald et al., 2011b; Weil et al., 2016).

Reduction of contrast sensitivity has been found to be one of the earliest signs of PD suggesting that this defect could be used as a premotor biomarker (Guo et al., 2018). Patients with normal visual acuity but with a loss in contrast sensitivity have been described (Regan and Neima, 1984). Moreover, this deficit is progressive and correlates with PD disease progression (Diederich et al., 2002; Miri et al., 2016; Ridder et al., 2017). Changes in contrast sensitivity in PD is partly reversible by the administration of the DA precursor levodopa (L-DOPA) and have been associated with DA depletion in the retina and loss of cells in the GCL (Bulens et al., 1987; Hutton et al., 1993; Polo et al., 2016). However, the loss of contrast sensitivity seems to be dependent on the orientation of the stimulus indicating an involvement of higher visual centers (Weil et al., 2016).

Impaired color vision is one of the most prevalent visual dysfunctions observed in PD patients, correlates with the progression of the disease, and may represent a specific non-motor feature of PD (Buttner et al., 1994; Diederich et al., 2002; Muller et al., 2002; Postuma et al., 2006; Piro et al., 2014). It has been shown that color vision abnormalities can be present several years before PD diagnosis suggesting that this defect could be an early PD biomarker (Buttner et al., 1995; Postuma et al., 2006; Diederich et al., 2010). Notably, deficits of color discrimination have also been reported in patients with rapid eye movement (REM) and sleep behavior disorder (RBD) (Postuma et al., 2006), which is considered as an early manifestation of α-synucleinopathies (Iranzo et al., 2013). Interestingly, PD patients with leucine-rich repeat kinase (*LRRK2*) gene mutations show a more severe impairment of color discrimination compared with idiopathic PD patients (Marras et al., 2011). However, discordant data have been reported indicating that color vision may not be consistently impaired in early PD (Vesela et al., 2001). It has been suggested that defective color vision may represent an early sign of DA dysfunction in PD (Piro et al., 2014; Armstrong, 2015) that can be ameliorated by L-DOPA administration (Buttner et al., 1994). However, besides DA dysfunction, the loss of cells in the GCL (Polo et al., 2016), the cognitive impairment and the involvement of dysfunctional cortical areas (Brandies and Yehuda, 2008) may also be associated with this defect, therefore suggesting that color vision abnormalities in PD seem to be due to multifactorial causes.

## Pathological Alterations in the Retina of PD Patients

In recent years, the development of non-invasive studies such as imaging of the retina and electrophysiological assessments allowed the direct observation of structural and functional changes in the retina in PD patients.

Optical coherence tomography (OCT) allows measurements of retinal layers *in vivo*, providing structural information of the retina with 1-to-10 micrometer (μm) resolution (Ang et al., 2018). In particular, OCT was used to analyze the peripapillary retinal nerve fiber layer (RNFL) and retinal thickness (i.e., macular volume scans). Different studies have shown a significant reduction in RNFL thickness of PD patients especially in the temporal quadrant (Inzelberg et al., 2004; Kirbas et al., 2013; La Morgia et al., 2013; Moreno-Ramos et al., 2013; Bodis-Wollner et al., 2014b; Lee et al., 2014a; Satue et al., 2016; Aydin et al., 2018; Matlach et al., 2018) that, notably, is typically affected in mitochondrial optic neuropathies (La Morgia et al., 2013; Maresca et al., 2013). In contrast, only a few studies failed to find differences between PD patients and healthy controls (Aaker et al., 2010; Archibald et al., 2011a; Albrecht et al., 2012; Tsironi et al., 2012). OCT analysis reported pathological thinning of the RGC, IPL and INL, more evident in the foveal pit zone (Cubo et al., 2010; Shrier et al., 2012; Adam et al., 2013; Spund et al., 2013; Bodis-Wollner et al., 2014b; Lee et al., 2014b). However, also in this case, other studies failed to find significant differences in PD patients (Aaker et al., 2010; Archibald et al., 2011a; Albrecht et al., 2012). These discrepancies can be attributed to differences in disease stage/severity, and to diverse measurement protocols and OCT equipment and analysis methods.

Electrophysiology techniques such as electroretinography (ERG) and visual evoked potentials (VEP) allow the analysis of selective retinal circuits and the determination of dysfunction of specific retinal cell types.

A close electrophysiological examination of the retina has shown indeed a genuine impairment at the level of the local circuitry in PD subjects. ERG measures the electrical response evoked by a brief visual stimulus recorded from the retina. The analysis of the resulting electrical potential gives information about the function of different retinal cell types including cones, rods, photoreceptors and retinal interneurons (Creel, 1995). ERG is a very useful tool in order to obtain diagnostic information and for disease progression monitoring. Taking advantage of this technique, alterations in PD patients’ retina have been described. In particular, scotopic and photopic b-wave as well as the amplitude of the photopic a-wave have been found to be reduced (Gottlob et al., 1987; Burguera et al., 1990). Interestingly, a reduction of the amplitude of b-waves in PD patients is present at early stages of the disease (Ikeda et al., 1994; Nowacka et al., 2015) (Figure 2B). An increase in the latency of the VEP onset is another frequent finding in PD patients (Tartaglione et al., 1987; Liu et al., 2017; He et al., 2018).

However, it is necessary to remember that by recording at the level of the visual cortex, VEP does not provide information exclusively about the retina but rather about the whole visual pathway. To date, the precise mechanisms responsible for the alterations described above are not fully understood. It has been suggested that decreased levels of DA can account (at least in part) for these abnormalities. In agreement with this suggestion, lower levels of TH or DA in the retina of PD patients have been also described (Nguyen-Legros, 1988; Harnois and Di Paolo, 1990).

Different studies have demonstrated that PD patients display ERG profiles in which the amplitude and the latency are altered as compared to control age-matched subjects (Gottlob et al., 1987; Tartaglione et al., 1987; Burguera et al., 1990; Ikeda et al., 1994). Interestingly, the ERG alterations observed in PD patients can be mimicked with the DA receptor D2 antagonist I-sulpiride both in humans (Stanzione et al., 1995) and monkeys (Tagliati et al., 1994). As mentioned before, D2 receptor activation mimics the low levels of DA release. Although very informative, it is necessary to stress that these studies have important limitations since they provide only descriptive observations, without information on the cellular and molecular basis underlying the observed ERG impairment. Moreover, most of these studies focused on heterogeneous groups of patients thus making it difficult to extrapolate conclusive information.

These limitations in terms of mechanistic issues are compensated by the high clinical relevance that the combination of these ophthalmic diagnostic tools (i.e., ERG, PERG, OCT) may represent for early and non-invasive diagnosis of PD.

## Visual and Retinal Abnormalities in PD Animal Models

To date a limited number of studies has been performed on the retina of PD animal models; all these studies, however, consistently report visual and retinal abnormalities as summarized in Table 3.

**TABLE 3:** Retinal abnormalities in PD animal models.

	**Model**	**Biochemical and histological abnormalities**	**Visual defects**	**References**
*Rotenone*	Rat	Decreased number of RGCs and DA amacrine cells. Reduced thickness of INL and ONL	Decreased scotopic and photopic a- and b-waves	Biehlmaier et al., 2007; Esteve-Rudd et al., 2011; Normando et al., 2016
*6-OHDA*	Rat	Decreased dopamine level	NA	Meng et al., 2012
	Mouse	Decreased number of DA amacrine cells.	Decreased visual-acuity	Marrocco et al., 2020
	Monkey	NA	Abnormal PERG responses	Bodis-Wollner and Tzelepi, 1998
*MPTP*	Mouse	Decreased number of DA amacrine cells.	Reduction of oscillatory potentials and of b-wave	Takatsuna et al., 1992
	Monkey	RNFL thinning and a decreased macula volume and foveal thickness. Decreased number of DA, γ-aminobutyric acidergic and glycinergic amacrine cells.	Abnormal PERG and ERG responses Decreased visual acuity and contrast sensitivity	Bodis-Wollner and Tzelepi, 1998; Cuenca et al., 2005
*alpha-synuclein*	TgM83 (Prnp- A53T- SNCA) Mouse	Accumulation of pSer129-α-Syn in the outer retina. Increased microglial activation and GFAP immunoreactivity	NA	Mammadova et al., 2019
	Thy-1-A30P- SNCA Mouse	Accumulation of pSer129-α-Syn in GCL, IPL, and INL.	NA	Veys et al., 2019
	AAV-mediated wt hu-α-Syn overexpression	Accumulation of pSer129-α-Syn in GCL, IPL and INL. Decreased number of DA amacrine cells and of RGC.	Decreased b-wave in light-adapted condition. Decreased visual acuity	Marrocco et al., 2020
	α-Syn over-expression *Drosophila*	NA	Decrease ERG depolarization amplitude	Chouhan et al., 2016
*LRRK2*	LRRK2-G2019S *Drosophila*	Neurodegeneration in the retina	Decrease in the peak-to-peak amplitude of the ERG	Hindle et al., 2013
*DJ-1*	*DJ-1*α^Δ^ *^72^* and *DJ-1*β^Δ^ *^93^ Drosophila*	NA	Abnormal VEP	Himmelberg et al., 2018
*PINK-1*	*PINK1*^5^ *Drosophila*	NA	Abnormal VEP	Himmelberg et al., 2018

**NA, not analyzed.**

Ocular abnormalities were studied in the classical neurotoxin-induced rodent models generated by systemic administration or intracranial injection of drugs, such as Rotenone, 6-hydroxydopamine (6-OHDA), or 1-methyl- 4-phenyl-1,2,3,6-tetrahydropyridine (MPTP).

Rotenone is a naturally occurring pesticide and a potent inhibitor of the Mitochondrial Respiratory Chain (MRC) complex I and is used to model PD in animals. Rotenone-treated rats showed a decreased number of RGC and DA amacrine cells as wells as decreased thickness of the INL and ONL (Biehlmaier et al., 2007; Esteve-Rudd et al., 2011; Normando et al., 2016). These abnormalities were accompanied by a decrease in the amplitude of scotopic and photopic a- and b-waves (Esteve-Rudd et al., 2011). Notably, in this model, *in vivo* OCT analysis and detection of apoptotic retinal cells (DARC) demonstrated retinal neurodegeneration at 20-days post-rotenone injection, while degeneration of DA neurons in the SN and striatum became evident at day 60. These data indicate that neurodegeneration occurs first in the retina and then in the brain supporting the idea that the retina can be used as a potential biomarker tissue for early diagnosis.

6-OHDA and MPTP are toxins that selectively destroy DA containing neurons and are widely used to induce PD in animal models. Unilateral injection of 6-OHDA into the substantia nigra induced a decrease in DA levels in the retina of a rat PD model (Meng et al., 2012). Abnormal PERG responses of the RGC have been observed in monkeys intravitreally injected with 6-OHDA (Bodis-Wollner and Tzelepi, 1998). Finally, intravitreal administration of 6-OHDA in mice resulted in a significant decrease of DACs and impairment of visual-acuity, which was rescued by L-DOPA (Marrocco et al., 2020).

Injection of MPTP in mice and monkeys causes a reduction in the number of TH-positive amacrine cells and in retinal dopamine levels (Tatton et al., 1990; Cuenca et al., 2005). In MPTP-injected monkeys Cuenca and collaborators also reported a decrease in γ-aminobutyric acidergic and glycinergic amacrine cells, a deterioration of AII amacrine cells exhibiting a loss of lobular appendages and dendritic processes, abnormal electrical synapses among AII cells, as well as chemical synapses between these and rod bipolar cells (Cuenca et al., 2005). Moreover, similar to PD patients, MPTP monkeys also show RNFL thinning, a decreased macula volume and foveal thickness as shown by OCT (Schneider et al., 2014). These defects resulted in abnormal PERG and ERG responses and a decline in visual acuity and contrast sensitivity (Bodis-Wollner and Tzelepi, 1998).

Takatsuna and collaborators observed a reduction in the amplitude of oscillatory potentials and b-wave after intraperitonel injection of MPTP and ERG analysis 10 and 30 days after MPTP injections in C57BL/6J mice (Takatsuna et al., 1992). Primates treated both with MPTP or 6-OHDA (Ghilardi et al., 1988, 1989) showed spatial frequency-dependent abnormalities in both PERG and VEP, and L-DOPA administration was effective in rescuing the described ERG defects (Ghilardi et al., 1988, 1989; Bodis-Wollner and Tzelepi, 1998).

Besides the toxin-induced models, ocular abnormalities were also studied in animal models of PD including transgenic models overexpressing both wild-type (wt) and mutated α-Syn, *LRRK2* mutations, and knockout models of *PINK-1* and *DJ-1.*

Mammadova et al. (2019) analyzed TgM83 transgenic mice expressing A53T human α-Syn under the control of the mouse prion protein (Prnp) promoter and showed that α-Syn accumulates in the inner and outer retina of transgenic mice, while phospho-α-Syn was significantly increased in the ONL. The data highlighted a difference between this model and what has been found in PD patients’ retinas where detection of pSer129-α-Syn in the outer retina was not reported (Beach et al., 2014; Bodis-Wollner et al., 2014a; Ho et al., 2014; Ortuno-Lizaran et al., 2018). In addition, TgM83 transgenic mice showed increased microglial activation followed by increased GFAP immunoreactivity. No differences in retinal TH-positive cells were observed (Mammadova et al., 2019), however TH-positive cells were only analyzed in vertical slides which presents some limitations for a comprehensive quantification of the DACs with respect to whole mount retina analysis.

Similar to what has been observed in human PD retinas, the analysis of the (Thy-1)- hu-A30P- α-Syn transgenic mouse showed the expression of the α-SYN transgene in the inner retinal layers while phospho-α-Syn was found in INL cells, in the cell bodies of the GCL, and in neurites in the IPL (Veys et al., 2019). No experiments were performed in either study to assess visual functions.

**FIGURE 3:**
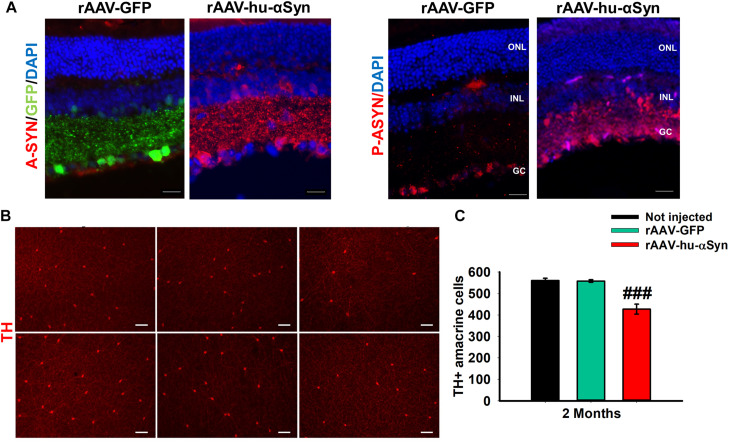
**(A)** Representative immunofluorescence showing retinal sections of rAAV-hu-α-syn injected mice and rAAV-GFP mice stained with antibody anti-hu-α-syn (A-SYN) (red) and antibody anti-phospho α-syn (P-ASYN). **(B)** Representative images of the retina whole mount anti-TH immunofluorescence on Not injected, rAAV-GFP- and rAAV-hu-α-syn-injected mice at 2 months post-injection. **(C)** Number of TH-positive cells. *N* ≥ 5. Data represent mean ± SEM. ###*p* < 0.0001 vs. rAAV-GFP. Significance was calculated by one-way ANOVA. Modified from Marrocco et al. (2020) under terms of the Creative Commons Attribution 4.0 International License (http://creativecommons.org/licenses/by/4.0/).

Recently, intravitreal injection of adeno-associated viral (AAV) vectors over-expressing the wt human α-Syn (hu-α-Syn) has been used to evaluate the effects of α-Syn over-expression in the mouse retina (Marrocco et al., 2020). AAV-hu-α-Syn injected mice displayed a time-dependent decrease of the amplitude of light-adapted responses while those elicited in dark-adapted conditions were not affected. Similarly, over-expression of the human hu-α-Syn in the retina of adult mice caused an impairment of the b-wave in light-adapted condition and a decreased visual acuity that was completely rescued by L-DOPA systemic administration. As for (Thy-1)-hu-A30P-α-Syn transgenic mice and PD patients’ retinas, pSer129-α-Syn accumulation were found in cells bodies of GCL and INL, and in the IPL neurites (Figure 3A). These results were mirrored by an early loss in the number of TH + amacrine cells (Figures 3B,C) that precede in time the loss of the RGCs (Marrocco et al., 2020). Notably this study demonstrated for the first time that α-Syn overexpression in the retina leads to neurodegeneration of DA amacrine cells, causing retinal-specific defects and consequent visual impairment that resemble the human PD phenotype (Marrocco et al., 2020). These findings is even more interesting in light of very recent evidence reporting a specific reduction of the DAC number as well as of the number of their synaptic contacts with AII amacrine cells and melanopsin cells reported in the retina of PD patients (Ortuno-Lizaran et al., 2020).

The genetic component of PD had been neglected for long time but the discovery of genes associated with this disorder has stressed the importance of this aspect in the pathogenesis of the disease. Mutations found in PD patients have been inserted in *Drosophila* and retinal functions have been examined. Among the mutations tested, one of the most common is the *LRRK2-*G2019S mutation. Its impact on visual function has been assessed by ERG following a 500 millisecond (msec) stimulation with blue light. Electrophysiological experiments revealed that compared to currents recorded from controls flies, the decrease in the peak-to-peak amplitude of the ERG observed in mutated flies was age-dependent, since it was detected starting at 10 days, reaching a minimal value at 28 days (Hindle et al., 2013). At the anatomical level, this mutation induced a marked neurodegeneration in the internal structure of the retina. Interestingly, this effect is not accompanied by loss of dopaminergic neurons (Hindle et al., 2013).

More recently, a comparison of visual function among *Drosophila* models carrying the early-onset PD mutations *DJ-1*α^Δ^ *^72^, DJ-1*β^Δ^ *^93^*, and *PINK1*^5^ (Himmelberg et al., 2018) has been performed. In order to investigate the consequences of the above mutations on the response of neuronal populations in the retina, the authors recorded steady-state VEP (SSVEP) from the surface of the *Drosophila* eye following stimulation with a sequence of frequency-tagged flickering stimuli. By using this experimental paradigm, the authors reported an abnormal increase in the SSVEP amplitude induced by all three mutations (Himmelberg et al., 2018). The *Rh1-GAL4* driver has been used in order to achieve over-expression of the full-length α-Syn protein in *Drosophila*. This manipulation was responsible for a mild decrease of ERG depolarization amplitude according to an age-dependent model reaching the strongest effect at 30 days (Chouhan et al., 2016).

## Molecular Mechanisms Implicated in PD Visual Abnormalities

Visual dysfunction in PD patients may be mainly explained by the depletion of DA due to the loss of amacrine and inner plexiform cells of the retina. Notably, changes in both contrast sensitivity and color vision are partly reversible by L-DOPA administration (Bulens et al., 1987; Hutton et al., 1993; Buttner et al., 1994). Moreover, changes in DA levels and depletion of amacrine cells lead to alterations in the receptive properties of RGCs, which eventually results in additional dysfunction of visual processing in PD patients. Nevertheless, electrophysiological tests and structural imaging point to defects in all inner retinal layers (GCL, IPL, and INL), indicating a loss of RGCs together with the loss of the dopaminergic plexus (Cubo et al., 2010; Shrier et al., 2012; Adam et al., 2013; Spund et al., 2013; Beach et al., 2014; Bodis-Wollner et al., 2014b; Lee et al., 2014b). This is in line with the finding of α-Syn aggregates in the GCL, IPL, and INL in the retina of both PD patients and animal models that may indeed represent one of the main causes of visual abnormalities in PD.

It has been shown that α-Syn toxicity impacts multiple pathways and impairs the functions of several organelles as well as inter-organelle contacts and organelles axonal transport. Normally α-Syn localizes to presynaptic termini and associates with synaptic vesicles (Iwai et al., 1995; Kahle et al., 2000). Different studies showed that the normal function of α-Syn might be disrupted in synucleinopathies, resulting in impaired synaptic-vesicle motility and decreased synaptic-vesicle recycling-pool size (Masliah et al., 2000; Nemani et al., 2010; Choi et al., 2013; Janezic et al., 2013). Moreover, DA terminal loss, deficient DA release, reduction in dopamine re-uptake and defective DAT function have been also shown indicating that abnormal α-Syn can disrupt dopamine turnover through different mechanisms (Masliah et al., 2000; Lundblad et al., 2012; Janezic et al., 2013; Giordano et al., 2018). On the other hand, α-Syn toxicity also causes dysfunctions of different organelles including the endoplasmic reticulum and the Golgi, autophagy or lysosomal pathways, and mitochondria (reviewed in Wong and Krainc, 2017). Recently, an important crosstalk between α-Syn and mitochondrial disfunctions has been described. α-Syn toxicity can indeed directly disrupt mitochondrial homeostasis through different mechanisms (Wong and Krainc, 2017; Vasquez et al., 2020) such as deregulation of mitochondrial dynamics, including fission/fusion and mitophagy processes (Kamp et al., 2010; Choubey et al., 2011; Nakamura et al., 2011; Chen et al., 2015; Ordonez et al., 2018), as well as damage to the mitochondrial DNA (mtDNA) and impaired mitochondrial protein import (Martin et al., 2006; Di Maio et al., 2016). Moreover, α-Syn toxicity may also induce mitochondrial dysfunction indirectly by decreasing the level of PGC-1α, a key player in mitochondrial biogenesis (Zheng et al., 2010; Eschbach et al., 2015).

These finding point to a pivotal role of mitochondrial dysfunction in PD pathogenesis. Notably, in a recent study it was shown that AII cells were not reduced in PD patients, but they showed the loss of mitochondria in lobular appendages, which may indicate an energetic failure, and a loss of connexin 36, suggesting alterations in the AII coupling and in visual signal transmission from the rod pathway (Ortuno-Lizaran et al., 2020).

Moreover, mitochondrial defects can represent *per se* a cause of PD. The first observation involving mitochondria in PD was the discovery that MPTP-induced parkinsonian syndrome is due to MPTP-dependent inhibition of MRC complex I (Davis et al., 1979; Langston et al., 1983; Ramsay and Singer, 1986). MPTP is metabolized to MPP + by MAO-B in glial cells and selectively concentrates in dopaminergic neurons through the dopamine transporter (DAT) (Javitch et al., 1985). As we have seen before, most of the toxins that leads to loss of DA cells and associated parkinsonism act either as Complex I inhibitors, such as Rotenone, or lead to increased production of reactive oxygen species (ROS) such as Paraquat and 6-OHDA (Betarbet et al., 2000; Jenner, 2001; Blesa et al., 2012; Giannoccaro et al., 2017). Moreover, many genes associated with PD encode proteins that impact on mitochondrial function and clearance (Exner et al., 2012) (see Table 1). Lastly, bioenergetics defects and decreased activity of MRC complexes (in particular Complex I) have been found in brains and peripheral tissues of idiopathic PD patients (Chaturvedi and Flint Beal, 2013; Bose and Beal, 2016). Of note, in many cases this may be the result of genetic predisposition, possibly related to the mtDNA, considering the occurrence of mutations, deletions, and defective mtDNA maintenance with a reduction of copy number, as a major driving mechanism of PD (Giannoccaro et al., 2017).

Interestingly, OCT analysis in PD patients reported a reduction of the RNFL thickness and a significant thinning of nerve fibers entering the infero-temporal quadrants of the optic disk, consistent with the involvement of the papillo-macular bundle (Inzelberg et al., 2004; Yavas et al., 2007; Moschos et al., 2011; La Morgia et al., 2013). This pattern of axonal loss is similar to that typically seen in Leber hereditary optic neuropathy (LHON) and in dominant optic atrophy (DOA), the most frequent mitochondrial optic neuropathies, where the temporal fibers belonging to the papillo-macular bundle are specifically susceptible (Maresca et al., 2013; Yu-Wai-Man et al., 2016). Notably, both LHON and DOA are associated with MRC Complex I defects (Yu-Wai-Man et al., 2011), which is also recognized as a key feature in the pathogenesis of sporadic and genetic forms of PD (Giannoccaro et al., 2017). Moreover, DOA, LHON, and PD are also associated with altered mitochondrial dynamics. Mitochondrial network fragmentation has been described in patient-derived cells from both DOA and PD affected individuals carrying, respectively, OPA1 and PINK1/Parkin mutations (Schapira, 2008; Carelli et al., 2009; Whitworth and Pallanck, 2009). Further, deregulation of the mitochondrial quality control mechanisms and mitophagy has been documented in RGCs of OPA1 mutant mice, and represents a key factor in PD pathogenesis (White et al., 2009; Exner et al., 2012; Giannoccaro et al., 2017).

These common mechanisms can explain the similar retinal phenotype seen in patients with PD and those with LHON and DOA, suggesting that they might also share similar therapeutic targets. Strategies that enhanced mitophagy and mitochondrial biogenesis resulted indeed in an amelioration of the phenotype in both LHON and PD representing attractive targets for drug development (Zheng et al., 2010; Dikic and Bremm, 2014; Eschbach et al., 2015; Koentjoro et al., 2017; Indrieri et al., 2019, 2020).

## Conclusion and Future Perspectives

Visual abnormalities together with impairment of the retinal dopaminergic system is an intriguing phenotype in PD patients and animal models. The retina, however, has long been overlooked compared to other brain regions in PD, so the information available is rather limited. However, recent experimental evidence indicates ocular changes in PD as promising biomarkers in the eye that can be potentially used for early diagnosis, to track disease progression, and to evaluate novel therapeutic strategies (Guo et al., 2018; Turcano et al., 2019; Veys et al., 2019). Although the specificity and predictive value of OCT and ERG changes in PD patients are still under debate, due to overlap with normal aging and other neurological and ophthalmological diseases, a better characterization of retinal dysfunctions during neurodegenerative diseases combined with imaging of protein aggregates may indeed represent a valuable approach for early diagnosis of PD. In this respect the presence of α-Syn deposits in the retina may have high potential for early diagnosis of PD (Veys et al., 2019; Ortuno-Lizaran et al., 2020). We thus believe that novel approaches integrating multiple biomarkers and employing novel technologies to increase diagnostic yield are needed and may be successfully applied in PD.

Moreover, due to its anatomical organization, the retina could be an ideal structure to study the molecular mechanisms underlying PD pathology. The retina-model of PD does not replace the nigrostriatal models of PD, as it cannot recapitulate the cognitive and motor deficits as well as the complexity of the synaptic changes occurring in the mesocorticolimbic system in PD. However, it could have some advantages that combine those of *in vivo* brain models and *in vitro* cellular models. The advantage of using the retina as compared to *in vitro* models (including IPS or organoids) is that the animal retina recapitulates the retina of patients and the complexity of the neuronal populations present *in vivo*. Furthermore, *in vivo* studies on disease mechanisms or on drug screening on the retina allows to test vision. Differently from the standard *in vivo* mesencephalic models the retina in animals is accessible with non-invasive methods (such as OCT, ERG, and VEP) that allow a comprehensive analysis of the histological and functional progression of neuronal loss. This makes it particularly useful to follow the time-course of novel therapeutic strategies for months in the same subjects, which is one of the main limitations in animal models of neurodegeneration. Another important aspect is that it allows the local application of drugs, which is important for generating proof-of-concept evidence with novel pharmacological or genetic therapeutic approaches without using invasive (like intra-brain injection) and/or systemic injections that might have peripheral side effects. Therefore, the retina-model might be the first step for *in vivo* testing of disease modifiers. This aspect is also important for translating new therapeutic approaches to patients. Gene or pharmacological local treatments of the eyes are already at an advanced stage. There is the possibility of testing the efficacy of a treatment for a single patient by treating with local applications one single eye and evaluating it by *in vivo* imaging and functional tests. This would also contribute, together with studies on the patient IPS cells, to developing personalized therapies. For all these reasons we propose the retina as a useful complementary experimental model for the identification and study of pathways involved in the disease pathogenesis or to test novel therapeutic approaches for PD.

## Author Contributions

ED and AI conceived and wrote the first draft of the manuscript. RP wrote the electrophysiological part of the manuscript. BF edited the manuscript. All authors shaped the final version of the manuscript.

## Conflict of Interest

The authors declare that this study received funding from Fondazione Roche. The funder was not involved in the study design, collection, analysis, interpretation of data, the writing of this article or the decision to submit it for publication.
